# Isorhamnetin Attenuates Atherosclerosis by Inhibiting Macrophage Apoptosis via PI3K/AKT Activation and HO-1 Induction

**DOI:** 10.1371/journal.pone.0120259

**Published:** 2015-03-23

**Authors:** Yun Luo, Guibo Sun, Xi Dong, Min Wang, Meng Qin, Yingli Yu, Xiaobo Sun

**Affiliations:** 1 Key Laboratory of Bioactive Substances and Resources Utilization of Chinese Herbal Medicine, Ministry of Education, Institute of Medicinal Plant Development, Chinese Academy of Medical Sciences & Peking Union Medical College, Beijing, PR China; 2 Academy of Chinese Materia Medica, Wenzhou Medical University, Wenzhou, Zhejiang, China; Max-Delbrück Center for Molecular Medicine (MDC), GERMANY

## Abstract

**Background and Purpose:**

Isorhamnetin (Iso) is a flavonoid compound extracted from the Chinese herb *Hippophae rhamnoides* L. Previous studies have revealed its anti-cancer, anti-inflammatory, and anti-oxidant activities. This study investigated the ability of Iso to inhibit oxidized low-density lipoprotein (ox-LDL)-induced cell apoptosis in THP-1-derived macrophages. The effects of Iso on atherosclerosis *in vivo* were also evaluated in apolipoprotein E knockout (ApoE-/-) mice fed a high fat diet.

**Methods and Results:**

Iso showed significant inhibitory effects on ox-LDL-induced THP-1-derived macrophage injuries via decreasing reactive oxygen species levels, lipid deposition, and caspase-3 activation, restoring mitochondrial membrane potential, reducing the number of terminal deoxynucleotidyl transferase-mediated dUTP nick end-labeling (TUNEL)-positive cells, and regulating apoptosis-related proteins. We also determined the protective effects of Iso by PI3K/AKT activation and HO-1 induction. Iso reduced the atherosclerotic plaque size *in vivo* in ApoE-/- mice as assessed by oil red O, Sudan IV staining, and CD68-positive cells, and reduced macrophage apoptosis as assessed by caspase-3 and TUNEL assays in lesions.

**Conclusion:**

In conclusion, our results show that Iso inhibited atherosclerotic plaque development in ApoE-/- mice by PI3K/AKT activation and HO-1 induction.

## Introduction

Atherosclerosis (AS), a chronic inflammatory disease characterized by the accumulation of lipids and fibrous elements in large arteries, is the most common type of coronary artery disease and the leading cause of morbidity and mortality worldwide [1]. Oxidized low density lipoproteins (ox-LDLs) and macrophages play vital roles in the pathogenesis of AS by promoting intracellular lipid accumulation and foam cell formation [2]. Studies have indicated that ox-LDL can be phagocytosed by macrophages via macrophage scavenger receptors, CD36, Toll-like receptors, and LOX-1 receptors [3], resulting in macrophage activation [4]. Subsequently, activated macrophages will produce reactive oxygen species (ROS) [5]. Excessive ROS production stimulates the detrimental modification of vital intracellular macromolecules, such as lipids, proteins, and DNA, resulting in macrophage apoptosis [6]. The induction of foam cell apoptosis is involved in subsequent plaque formation. The inhibition of ox-LDL-induced macrophage apoptosis and the modulation of intracellular ROS levels may be an attractive strategy to prevent and minimize the development of AS.

Heme oxygenase-1 (HO-1), one of the endogenous cytoprotective enzymes produced during in response to oxidative stress, has recently attracted considerable attention [7, 8]. HO-1 induction has been shown to generate effective antioxidant bilirubin and carbon monoxide and thus shows potential regulatory properties against cell oxidative injury [9]. Studies have also shown that the regulation of HO-1 expression is associated with the activation of nuclear erythroid-2-related factor (Nrf2) [10]. Under normal conditions, Nrf2 is bound to its repressor, Keap1, and is inactive in the cytoplasm. Under oxidative stress conditions, Nrf2 is liberated from the Keap1-Nrf2 complex and translocates to the nucleus [11]. The phosphatidylinositol 3-kinase /protein kinase B (PI3K/Akt) signal transduction pathway is involved in Nrf2 nuclear translocation [12]. Emerging studies have suggested that HO-1 provides cytoprotection against oxidative stress [13, 14]. Therefore, the activation of PI3K/AKT and the modulation of HO-1 expression may present a novel target for inhibiting ox-LDL-induced cell apoptosis.

**Fig 1:**
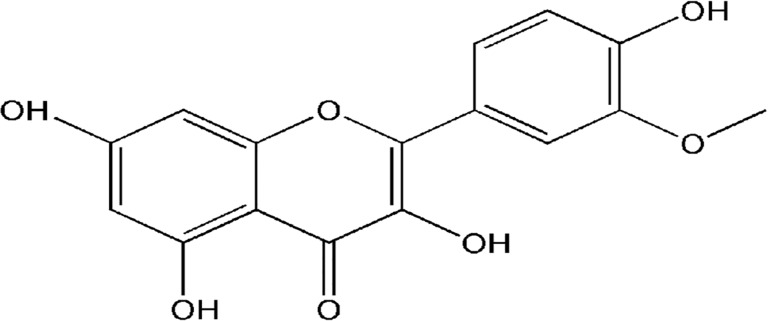
Chemical structure of Iso.

Isorhamnetin (Iso; Fig. 1) is a bioactive compound found in herbal medicinal plants, such as *Hippophae rhamnoides* L., *Oenanthe javanica* and *Ginkgo biloba* L., and possesses multiple biological properties. Our laboratory previously demonstrated that Iso inhibits the H_2_O_2_-induced activation of the intrinsic apoptotic pathway by scavenging free ROS and inactivating ERK [15] and protects against doxorubicin-induced cardiotoxicity *in vivo* and *in vitro* [16]. Bao and colleagues found that Iso had protective effects on ox-LDL-induced endothelial cell injuries by increasing antioxidant activity and activating p38MAPK signaling pathway [17]. Anti-tumor [18], antiviral [19], and anti-inflammatory properties [20] and neurodegenerative injury protection [21] have also been reported. These studies highlight the potential function of Iso in the prevention and treatment of cardiovascular and cancer diseases. However, the preventative or protective properties of Iso against AS and its underlying molecular mechanisms have yet to be defined.

In the present study, we investigated the protective effects and mechanisms of Iso on ox-LDL-induced macrophage apoptosis, oxidative stress, and AS in apolipoprotein E knockout (ApoE-/-) mice fed a high fat cholesterol (HFC) diet containing 20% fat and 0.3% cholesterol. We also determined the involvement of the classical PI3K/AKT signaling pathway and HO-1 induction in the anti- apoptotic properties of Iso.

## Materials and Methods

### Materials

Iso (molecular weight = 316.27; purity, 98%) was purchased from Shanghai Winherb Medical S&T Development (Shanghai, China). Lovastatin (LOV; purity, 99%) was acquired from the National Institutes for Food and Drug Control (Beijing, China). Ox-LDL was purchased from Peking Union-Biology Co., Ltd. (Beijing, China). Cell culture materials were purchased from GIBCO (Grand Island, NY, USA) and Hyclone (Logan, UT, USA). Fluorescent dye (JC-1) was obtained from Invitrogen (CA, USA). Total cholesterol (TC), triglyceride (TG), low-density lipoprotein cholesterol (LDL-C), and high-density lipoprotein cholesterol (HDL-C) commercial kits were purchased from Zhongshan Bio-tech Co., Ltd. (Beijing, China). Myeloperoxidase (MPO) activity colorimetric assay kit, caspase-3 activity and ROS fluorimetric assay kits were purchased from BioVision Inc. (CA, USA).Glutathione peroxidase (GSH-px) commercial assay kit was acquired from Nanjing Jiancheng Bioengineering Institute (Nanjing, China). NOX activity kit was provided by Beijing Solarbio Science & Technology Co., Ltd (Beijing, China). Terminal deoxynucleotidyl transferase-mediated dUTP nick end-labeling (TUNEL) assay kit was purchased from Roche Diagnostics GmbH (Mannheim, Germany). All antibodies were obtained from Santa Cruz Biotechnology, Inc. (CA, USA). Phorbol 12-myristate13-acetate (PMA), dimethylsulfoxide (DMSO), 3-(4,5-dimethylthiazol-2yl-)-2,5-diphenyl tetrazolium bromide (MTT), oil red O, Sudan IV, LY290042, znic protoporphyrin (ZnPP) and other chemicals were purchased from Sigma-Aldrich (St. Louis, MO, USA).

### Cell Culture and Treatment

The human monocyte leukemia cell line THP-1 was purchased from the Type Culture Collection of the Chinese Academy of Sciences (Shanghai, China), and cultured in RPMI-1640 medium supplemented with 10% (v/v) fetal bovine serum (FBS) and 100 U/ml penicillin-streptomycin. Cells were maintained in a humidified incubator at 37°C in a 5% CO_2_ atmosphere. THP-1 monocytes (1 × 10^6^ cells/ml) were differentiated to macrophages using 160 nM PMA with 10% FBS for 48 h [22]. The THP-1-derived macrophages were pretreated with various doses (5, 10, and 20 μM) of Iso for 8 h and then stimulated with ox-LDL for 24 h. Hanks’ balanced salt solution was used to remove the oxidant before analysis.

### Analysis of Cell Viability

Cell viability was measured using the MTT assay as previously described [23]. Briefly, THP-1 cells were seeded at 1 × 10^5^ cells/well in 96-well plates. The cells were stimulated by PMA, pretreated with various doses (5, 10, and 20 μM) of Iso for 8 h, and incubated with 100 μg/ml ox-LDL for an additional 24 h. Then, 1 mg/ml MTT solution was added to each well and incubated for 4 h at 37°C. The cell culture medium was removed, and DMSO was added to dissolve the insoluble MTT-formazan salt. The absorbance was measured at 570 nm on a microplate reader (Tecan, Männedorf, Switzerland).

### Oil Red O Staining and Quantification

THP-1-derived macrophages were cultured in chamber slides with different doses of Iso (5, 10, and 20 μM) for 8 h and then incubated with ox-LDL for 24 h. Cells were washed three times with PBS, and stained with oil red O according to previously described methods [24]. Briefly, cells were fixed with 4% (w/v) paraformaldehyde (10 min, room temperature). After the treatments, cells were stained with filtered oil red O solution (30 min, 60°C) and observed under a microscope (Olympus, Tokyo, Japan). Image-Pro Plus 6.0 (IPP 6.0) software was used to obtain the integrated optical density (IOD) of oil red O staining.

### Measurement of Mitochondrial Membrane Potential (MTP; *ΔΨm*) Using JC-1

JC-1 was used to detect MTP changes with Iso treatment. Cells were incubated in chamber slides, washed with PBS, and incubated with 2 μM JC-1 dye for 30 min at 37°C in the dark as suggested by the manufacturer’s instructions. Cells were then washed with PBS, and images of the labeled cells were obtained under a fluorescence microscope (DM4000B; Leica Wetzlar, Germany) [16, 25]. IPP 6.0 software was used to measure the IOD of red and green fluorescence.

### Analysis of Caspase-3 Activation

Caspase-3 activity was measured using a fluorimetric assay kit according to the manufacturer’s instructions. Briefly, cells were collected after all indicated treatments and incubated on ice with 50 μl of chilled lysate buffer for 10 min. Approximately 50 μl of 2× reaction buffer (containing 10 mM dithiothreitol) and 5 μl of caspase-3 substrate (DEVD-AFC, 1 μM) were added. The mixture was incubated at 37°C for 2 h. The fluorescence intensity was read in a microplate reader (SpectraFluor, TECAN, Sunrise, Austria) at excitation and emission wavelengths of 400 and 505 nm, respectively. The results are described as fold changes compared with the control group.

### Assessment of Intracellular ROS Production

The level of intracellular ROS production was determined using a total ROS detection kit according to the manufacturer’s instructions. Briefly, following all indicated treatments, cells were washed with a 1× washing buffer, and incubated with 100 μl of 5-(and-6)-carboxy-2′,7′-dichlorodihydrofluorescein diacetate (25 μM final concentration) in the dark at 37°C for 30 min. Cellular DCF fluorescence intensity was determined using a microplate reader at excitation and emission wavelengths of 495 and 529 nm, respectively. The ROS level is expressed as a percentage of the control.

### Measurement of Glutathione Peroxidase (GSH-px), Myeloperoxidase (MPO) and NOX activity in Macrophages

The cellular activities of GSH-px and MPO were measured using commercial assay kits according to the manufacturers’ instructions. In brief, cellular supernatants were collected and mixed with the reaction mixture containing an MPO assay buffer and MPO substrate for 2 h at 25°C. Then, a TNB reagent was added, followed by a stop mixture. Sample absorbances were read at 412 nm. The macrophages were harvested, ultrasonicated, and centrifuged at 1000 rpm for 10 min at 4°C. The collected supernatant was used to evaluate the GSH-Px activity as recommended by the provided protocols.

NOX activity was monitored according to the manufacture’s instructions. Briefly, after all treatment, cells were obtained and washed with PBS, then cells were lysed with lysis buffer containing protease inhibitor cocktail (Roche). Subsequently, cells were sonicated for 10 seconds on ice and centrifuged at 11100 g for 10 min at 4°C. Supernatant was discarded and mitochondria were obtained. The mitochondria were sonicated for 6 min on ice. The mitochondrial lysates were incubated with NADPH test mixture for 1 min at 37°C, then the optical density was measured at 600 nm by microplate reader (Tecan, Männedorf, Switzerland). The NOX activity is expressed as a percentage of the control.

### TUNEL Staining in THP-1-Derived Macrophages

DNA fragmentation of apoptotic cells was detected using a TUNEL kit according to the manufacturer’s instructions. Briefly, THP-1-derived macrophages were cultured on cover slips for 48 h. After drug and vehicle treatments, the cells were fixed in 4% paraformaldehyde solution in PBS for 30 min at room temperature. The cells were then incubated with a methanol solution containing 0.3% H_2_O_2_ for 30 min at room temperature to block endogenous peroxidase activity. Macrophages were treated with a permeabilizing solution (0.1% sodium citrate and 0.1% Triton X-100) for 2 min at 4°C, incubated in the TUNEL reaction mixture for 60 min at 37°C, and visualized by fluorescence microscopy (DM4000B; Leica Wetzlar, Germany). Apoptotic cells were counted from four randomly selected fields in each sample, and the counts are expressed as a percentage of the total number of cells.

### Western Blot Analysis

THP-1-derived macrophages treated with ox-LDL or Iso were harvested, and washed with PBS. Cytoplasmic and nuclear protein samples were separated by protein extraction kits containing 1% phenylmethylsulfonyl fluoride (CoWin Bioscience Co., Ltd., Beijing, China). The protein concentration was measured using a BCA kit (Pierce Corporation, Rockford, USA). Equal amounts of protein from each sample were separated by electrophoresis on 10% sodium dodecyl sulfate polyacrylamide gels, and transferred onto nitrocellulose membranes (Millipore Corporation, Bedford, MD, USA) in Tris-glycine buffer at 100 V for 1 h in an ice box. The membranes were blocked in 5% (w/v) non-fat milk powder in Tris-buffered saline containing 0.1% (v/v) Tween-20 (TBST) for 2 h at room temperature and incubated overnight with primary antibodies (1:200) at 4°C The membranes were washed with TBST, incubated with appropriate secondary HRP-conjugated antibodies (1:1000) for 2 h on a shaking table, and washed with TBST three times for 45 min. An enhanced chemiluminescence solution was used to develop the blots for 5 min. The results were observed using Image Lab software (Bio-Rad, USA). The different protein levels are expressed as a percentage of the control, as calculated by Gel-Pro Analyzer software.

### Animals and Experimental Protocols

Six-week-old male C57BL/6J mice were used as wild-type control animals, and ApoE-/- mice were provided by the Experimental Animal Center of the Medical Department of Peking University. All animal care procedures and interventions were performed in accordance with the Guidelines and Policies for Animal Surgery approved by the Animal Ethics Committee of Peking Union Medical College (, Beijing, China). All mice were maintained in a temperature-controlled facility (temperature: 22 ± 1°C; humidity: 60%) with a 14 h light/10 h dark photoperiod and free access to food and water. All mice were fed an HFC diet (containing 0.3% cholesterol and 20% fat) for eight weeks, and randomly assigned into four groups. The control group (n = 10) comprised 10 C57BL/6J mice. Thirty male ApoE-/- mice were divided into the model (n = 10), Iso (n = 10), and LOV-positive (n = 10) groups. Aseptic carboxymethylcellulose sodium (0.5%) was used as the vehicle to dissolve Iso and LOV powder. The four aforementioned groups were orally administered with the vehicle, vehicle, Iso (20 mg/kg, i.g.), or LOV (3 mg/kg, i.g.) daily for eight weeks.

### Atherosclerotic Lesion Analysis

Serial sections of the aortic valve and entire aortas were stained with oil red O and Sudan IV, respectively, as previously reported [26]. Briefly, serial 10-μm-thick sections with 40-μm intervals were stained with oil red O. The adipose tissue was removed. The entire aorta was dissected, opened longitudinally, and stained with Sudan IV. The tissues were observed under an upright metallurgical microscope (Leica, Germany), and images of plaque size and lesion area were analyzed using IPP 6.0 software.

### Analyses of Atherosclerotic Lesion Macrophage Accumulation and Apoptosis

To identify the degree of involvement of macrophages in the formation of atherosclerotic lesions, immunofluorescent assays were used to stain for CD68, a macrophage marker. Nuclear counterstaining was achieved with DAPI. To further investigate macrophage apoptosis in lesions, TUNEL assays were performed as previously described [27]. The sections that were stained with TUNEL and DAPI were also stained for macrophage cytoplasm by an immunofluorescent assay. The TUNEL-positive cells and macrophage-associated apoptotic cells and bodies were visually counted in five sections per mouse.

### Serum Measurements

For serum lipid analysis, mice were fasted 16 h and weighed before collecting blood samples from their inner canthus for serum preparation. Serum samples were separated by centrifugation and stored at -80°C prior to analysis. Serum concentrations of TC, HDL-C, LDL-C, and TG were detected by a Hitachi7600 Automatic Biochemistry Analyzer (Tokyo, Japan) according to the manufacturer’s instructions.

### Statistical Analysis

All data are expressed as the mean ± SD from at least three independent experiments. Statistical comparisons between different groups were assessed by one-way ANOVA or the Student-Newman-Keuls method with GraphPad Prism 5.0 software. The level of significance was set at *P*<0.05.

### Ethics Statement

This study was performed following the regulations of the Chinese Guide for the Care and Use of Laboratory Animals published by the United States National Institutes of Health Publication No. 85–23, revised 1996.

## Results

### Iso Attenuates Ox-LDL-induced Cell Apoptosis in THP-1 Macrophages

**Fig 2:**
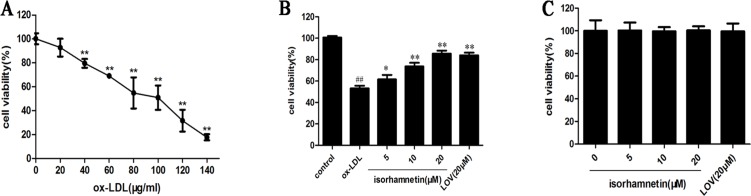
Protective effects of Iso against ox-LDL-induced THP-1-derived macrophage cell injury. THP-1 monocytes were incubated with PMA (160 nM) to induce differentiation into macrophages. (A) Effects of ox-LDL on macrophage cell viability. Cells were treated with increasing concentrations of ox-LDL (20, 40, 60, 80, 100, 120, and 140 μg/ml) for 24 h, and cell viability was determined by MTT assay. (B) Effects of Iso on ox-LDL-induced macrophage cell viability. Macrophages were pretreated with indicated doses of Iso and LOV for 8 h before exposure to 100 μg/ml ox-LDL for 24 h. Cell viability was measured by MTT assay. (C) Effects of Iso on macrophage cell viability. Cells were incubated with the indicated doses of Iso and LOV for 8 h. Cell viability was measured by MTT assay (A, B and C, expressed as the percentage of control). All results are expressed as the mean ± SD of three independent experiments. ^*##*^ *P* < 0.01 *vs*. control group; *P < 0.05, **P < 0.01 *vs*. ox-LDL-treated cells.

To investigate the protective effects of Iso on ox-LDL-induced macrophage apoptosis, we initially evaluated the general toxicity of ox-LDL. Cells were treated with 160 nM PMA for 48 h, followed by treatment with a series of ox-LDL concentrations (20, 40, 60, 80, 100, 120, and 140 μg/ml) for 24 h. Cell viability was measured using the MTT assay. As shown in Fig. 2A, cell viability was reduced to 50.92 ± 2.86% at 100 μg/ml ox-LDL compared with that in the control group; similar observations were made in a previous report [28]. Therefore, this study used 100 μg/ml ox-LDL in subsequent experiments.

The capacity for Iso to inhibit ox-LDL-induced macrophage injury was assessed. As shown in Fig. 2B, cell viability increased to 61.5 ± 2.1%, 73.6 ± 1.4%, and 85.6 ± 1.7% with pretreatment with different doses (5, 10, and 20 μM, respectively) of Iso compared with exposure to only 100 μg/ml ox-LDL for 24 h. LOV (20 μM), a positive control, significantly inhibited the cytotoxicity induced by ox-LDL treatment, and Iso (20 μM) showed a protective effect comparable with that induced by LOV. The cytotoxic effect of Iso on macrophages was subsequently measured. After treatment with various doses (5, 10, and 20 μM) of Iso, no change in cell viability was detected by MTT assay (Fig. 2C). These results indicate that Iso could protect THP-1-derived macrophages from ox-LDL-induced cell injury.

### Iso Suppresses Lipid Deposition

**Fig 3:**
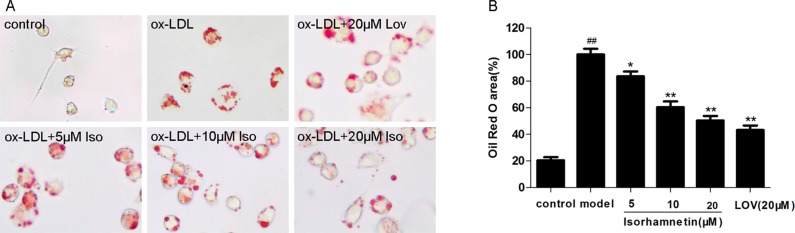
Iso reduced lipid deposition in THP-1-derived macrophages. THP-1-derived macrophages were pretreated with indicated doses of Iso for 8 h and then exposed to ox-LDL (100 μg/ml) for 24 h. (A) Representative images for each condition showing THP-1-derived macrophages stained with oil red O. (B) Area of the average size of lipid deposits. The results were obtained from three independent experiments. ^*##*^ *P* < 0.01 *vs*. control; *P < 0.05, **P < 0.01 *vs*. ox-LDL-treated cells.

Lipid deposition in macrophages promotes the development of AS [29]. To determine the effects of Iso on intracellular lipid levels, we performed oil red O staining. As shown in Fig. 3, ox-LDL exposure significantly increased lipid accumulation compared with the levels in the control group, whereas Iso treatment markedly decreased the lipid levels. Therefore, the oil red O staining results demonstrate that Iso significantly prevented intracellular lipid deposition in ox-LDL-induced macrophages.

### Iso Mitigates the Pro-Apoptotic Effects of Ox-LDL on Mitochondrial Injury, Caspase-3 Activation and TUNEL-Positive cells

**Fig 4:**
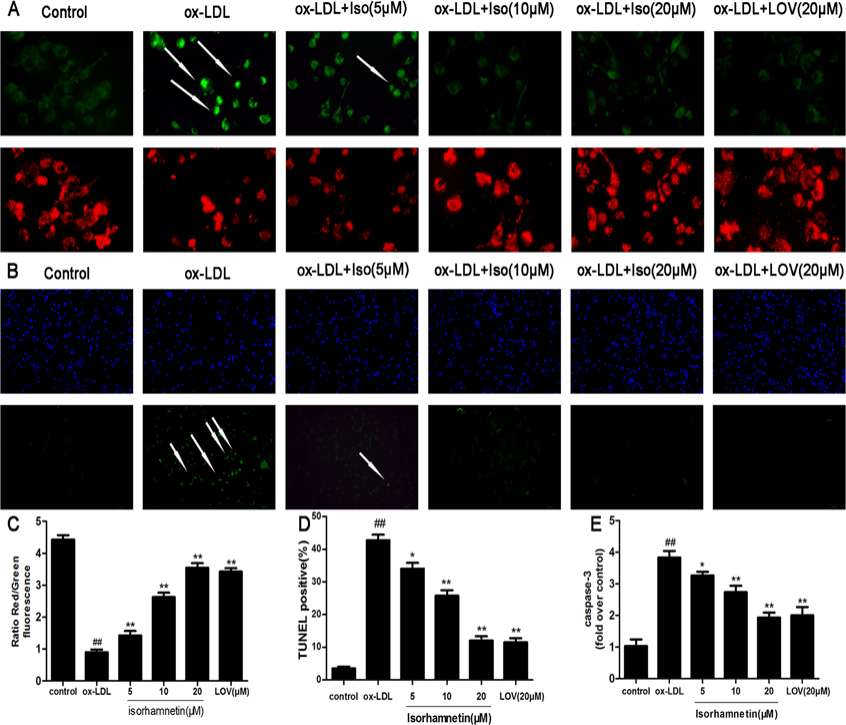
Iso mitigates the pro-apoptotic effects of ox-LDL on mitochondrial injuries, caspase-3 activation, and TUNEL-positive cells in THP-1-derived macrophages. THP-1-derived macrophages were pretreated with the indicated doses of Iso for 8 h and then exposed to ox-LDL (100 μg/ml) for 24 h. (A) Representative images showing macrophages stained with JC-1 dye as observed by fluorescence microscopy. (B) Internucleosomal DNA fragmentation was determined by TUNEL assay. (C) MTP in each group was calculated as the ratio of red to green fluorescence. (D) The TUNEL apoptotic index was determined by calculating the ratio of TUNEL-positive cells to total cells. (E) Caspase-3 activity was detected using a fluorimetric assay. Results are represented as the mean ± SD from three independent experiments. ^*##*^ *P* < 0.01 *vs*. control; *P < 0.05, **P < 0.01 *vs*. ox-LDL-treated cells.

MTP depolarization and caspase-3 cleavage are key processes involved in apoptosis [30]. To study the effects of Iso on MTP, the JC-1 assay was used to assess mitochondrial depolarization. Our results show that MTP was restored in cells pretreated with Iso compared with MTP in cells exposed to ox-LDL (Fig. 4A and 4C). Similarly, we found that Iso pre-incubation before exposure to ox-LDL could significantly protect ox-LDL-induced macrophage apoptosis, which was demonstrated by the reduced number of TUNEL-positive cells (Fig. 4B). Moreover, Fig. 4E shows that ox-LDL could significantly increase the activation of caspase-3, whereas pre-incubation with increasing doses of Iso could reduce caspase-3 activity. The restored MTP, decreased caspase-3 activity, and reduced number of TUNEL-positive cells suggest the anti-apoptotic effects of Iso.

### Iso Prevents Ox-LDL-Induced Oxidative Stress in THP-1-Derived Macrophages

**Fig 5:**
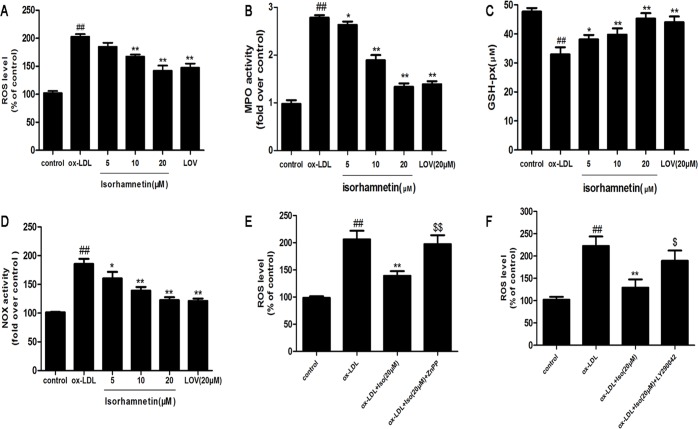
Iso prevents ox-LDL-induced THP-1-derived macrophage oxidative stress injuries. THP-1-derived macrophages were pretreated with the indicated doses of Iso for 8 h and then exposed to ox-LDL (100 μg/ml) for 24 h. (A) The intracellular ROS levels were measured with a fluorimetric assay. (B) MPO activity was determined by a colorimetric activity assay kit. (C) The GSH-px activity was measured using a commercial kit. (D) NOX activity was tested by a commercial kit. (E, F) THP-1-derived macrophages were pre-incubated with 10 μM ZnPP for 48 h or 20 μM LY290042 for 30 min, and then pretreated with Iso (20 μM) for 8 h followed by ox-LDL (100 μg/ml) for 24 h. Results are represented as the mean ± SD from three independent experiments. ^*##*^ *P* < 0.01 *vs*. control; *P < 0.05, **P < 0.01 *vs*. ox-LDL-treated cells; ^*$*^ *P <* 0.05, ^$$^ *P < 0*.*01 vs*. ox-LDL and Iso-treated cells.

A previous study demonstrated that ox-LDL increases the level of ROS, leading to cell apoptosis [6]. Therefore, we investigated ROS generation in response to ox-LDL stimulation and its regulation by Iso in THP-1-derived macrophages. As shown in Fig. 5A, the exposure to ox-LDL resulted in a remarkable increase in ROS production. Pretreatment with Iso significantly decreased intracellular ROS production. MPO and GSH-px activities are also indicators of the levels of oxidative stress. Thus, we measured the MPO and GSH-px activities. As shown in Figs. 5B and 5C, Iso inhibited the ox-LDL-induced up-regulation of MPO activity and increased the ox-LDL-induced down-regulation of GSH-px activity. As shown in Figs. 5D, NOX activity was significantly inhibited by Iso compared with ox-LDL treated group.

For further study, we tested the ROS production, as shown in Fig. 5E and 5F, the inhibitory effects of Iso was markedly reversed by the AKT and HO-1 inhibitors. These results suggest that Iso may modulate the ROS-generating enzyme, resulting in anti-oxidative effects. Altogether, Iso modulated ROS production and the ROS-related enzymes to reduce macrophage injuries.

### Iso Modulates the Expression of Apoptosis-Related Proteins

**Fig 6:**
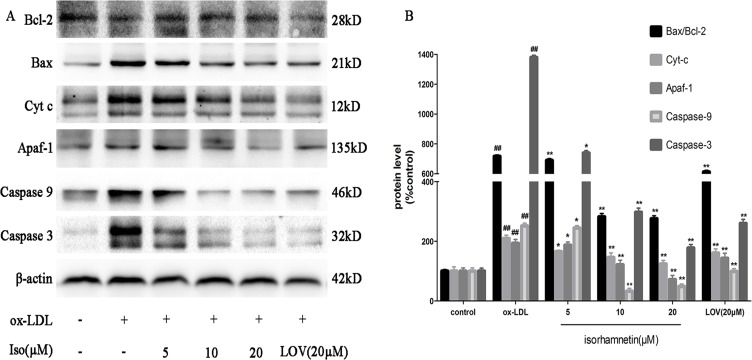
Iso modulates the expression of apoptosis-related proteins. After the indicated treatments, cells were harvested and lysed to measure Apaf-1, Cyt-c, Bcl-2, Bax, caspase-3, and caspase-9, and β-actin protein levels by western blot analysis. The blots are representative of three independent experiments, and values are presented as the mean ± SD from three independent experiments. ^*##*^ *P* < 0.01 *vs*. control; *P < 0.05, **P < 0.01 *vs*. ox-LDL-treated cells.

To explore the molecular mechanism of Iso treatment on the regulation of cell apoptosis, a western blot assay was performed. First, we studied the regulatory effects of Iso on apoptosis-related proteins, which were involved in ox-LDL-induced macrophage apoptosis [31]. As shown in Fig. 6, pretreatment with Iso reversed the effects of ox-LDL by decreasing Bcl-2 and increasing Bax expression levels. Similarly, ox-LDL treatment increased the contents of cytosolic Cyt-c and Apaf-1, but the production of caspase-3 and caspase-9 were obviously inhibited by Iso pretreatment. Accordingly, these results indicate that Iso could increase the up-regulation of anti-apoptotic proteins and decrease the down-regulation of pro-apoptotic proteins.

### Iso Exerts Its Effects by Activating the PI3K/AKT Pathway and Up-regulating HO-1 Expression

**Fig 7:**
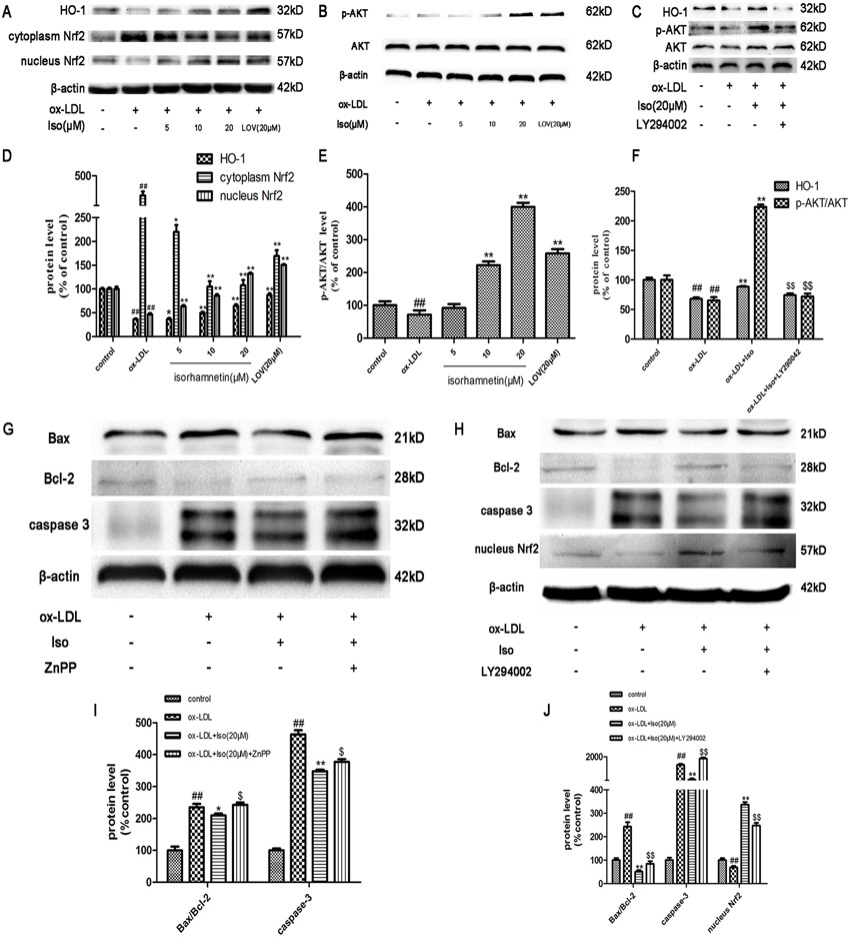
Iso activates PI3K/AKT and HO-1 induction in THP-1-derived macrophages induced by ox-LDL. THP-1-derived macrophages were exposed to ox-LDL (100 μg/ml) in the presence or absence of Iso (5, 10, and 20 μM) for 8 h. Expression levels of AKT, p-AKT, HO-1, and Nrf2 in the cytoplasm and nucleus were investigated by western blot. (A, D) Representative images of HO-1 and Nrf2 expression levels and statistical results relative to control group. (B, E) Representative images of p-AKT/AKT expression level and statistical results relative to control group. (C, F) Representative images of HO-1 and p-AKT/AKT expression level and statistical results relative to control group. (G, H, I, J) Representative images of Bax, Bcl-2, caspase-3, nucleus Nrf2 and β-actin expression level and statistical results relative to control group. The blots are representative of three independent experiments, and values are presented as the mean ± SD from three independent experiments. ^*##*^ *P* < 0.01 *vs*. control; *P < 0.05, **P < 0.01 *vs*. ox-LDL-treated cells; ^*$*^ *P <* 0.05, ^$$^ *P* < 0.01 *vs*. ox-LDL and Iso-treated cells.

HO-1 is a major component that defends against oxidative stress and is modulated by the nuclear accumulation of Nrf2. First, using western blot analysis, we evaluated the effect of Iso on inducing HO-1 expression and the nuclear accumulation of Nrf2. In Fig. 7A and 7D, THP-1-derived macrophages incubated with Iso showed a concentration-dependent increase in HO-1 protein expression and Nrf2 accumulation in the nucleus. However, the anti-apoptosis effects were dramatically reversed by HO-1 inhibitor, as shown in Fig. 7G and 7I. The PI3K/AKT pathway has been implicated in multiple cellular processes, including cell proliferation and survival. The phosphorylation of AKT was detected after exposure to Iso. As shown in Fig. 7B and 7E, Iso activated the phosphorylation of AKT in a concentration-dependent manner.

To further confirm the critical function of the PI3K/AKT pathway in the anti-apoptotic effects of Iso pretreatment, the PI3K inhibitor LY294002 was used. Iso induced HO-1 expression, and AKT phosphorylation was abolished by LY294002 (Fig. 7C and 7F).

As shown in Figs. 7H and 7J, Nrf2 translocation and the anti-apoptosis effects of Iso were significantly reversed by LY294002.

### Iso Relieves AS in ApoE^-^/^-^ Mice

**Fig 8:**
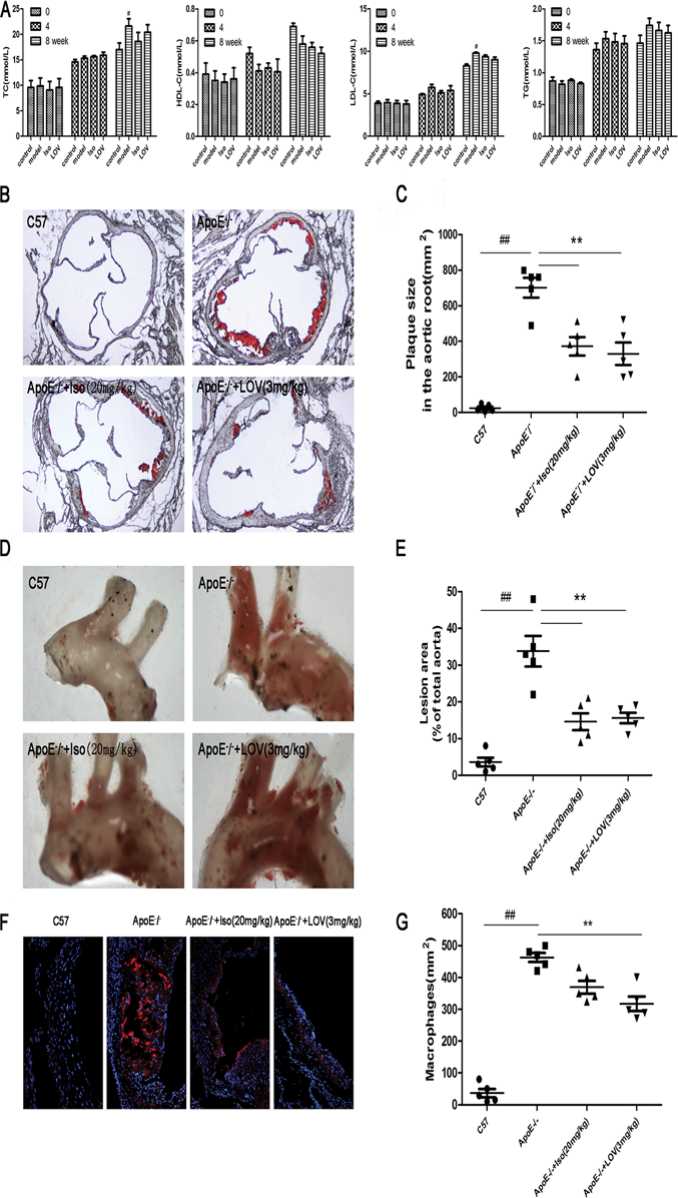
Iso relieves AS in ApoE-/- mice. All mice were fed HFC. ApoE-/- mice were treated with Iso (20 mg/kg, i.g.), LOV (3 mg/kg, i.g.), or its vehicle (as model group) for eight weeks; C57 mice were used as the control group. (A) Levels of serum lipids (TC, HDL-C, LDL-C, and TG) in mice at 0, 4, and 8 weeks of administration. (B, C) Representative light photomicrographs and morphometric analysis of oil red O-stained sections from the aortic root. (D, E) Representative images of Sudan IV-stained *en face* preparations of the proximal aorta and morphometric analysis of aortic AS expressed as a fraction of the total aortic area. (F, G) Representative images and morphometric analysis of CD68 staining from the aortic root. Values (*n* = 10 per group) are expressed as the mean ± SD. ^##^ *P* < 0.01 compared with the C57 control group; *P < 0.05, **P < 0.01, compared with the vehicle-treated ApoE-/- model group.

To examine the potential for Iso to inhibit the process of atherosclerotic lesions, blood lipid levels were measured. In addition, Oil red O staining of the aortic valve and Sudan IV staining of entire aortas were performed. As shown in Fig. 8A, no significant difference was found in the TC, TG, HDL-C, and LDL-C levels in ApoE-/- mice fed HFC only compared with those in the control, Iso, and LOV groups. However, the quantification of oil red O staining showed significant increases in lesion sizes in ApoE-/- mice compared with those in the control group; Iso was observed to reduce the sizes of the atherosclerotic lesions (Fig. 8B and 8C). Similarly, Sudan IV staining of the aorta showed a marked decrease in the total lesion area in the Iso group compared with that in ApoE-/- mice (Fig. 8D and 8E). Macrophage accumulation is a feature of atherosclerotic plaque [32]; CD68 immunofluorescence analysis was used to detect macrophage accumulation in lesions. The results show that Iso decreased macrophage accumulation in atherosclerotic lesions (Fig. 8F and 8G). Taken together, these results showed that Iso was able to relieve AS.

### Iso Decreases Macrophage Apoptosis in Atherosclerotic Lesions

**Fig 9:**
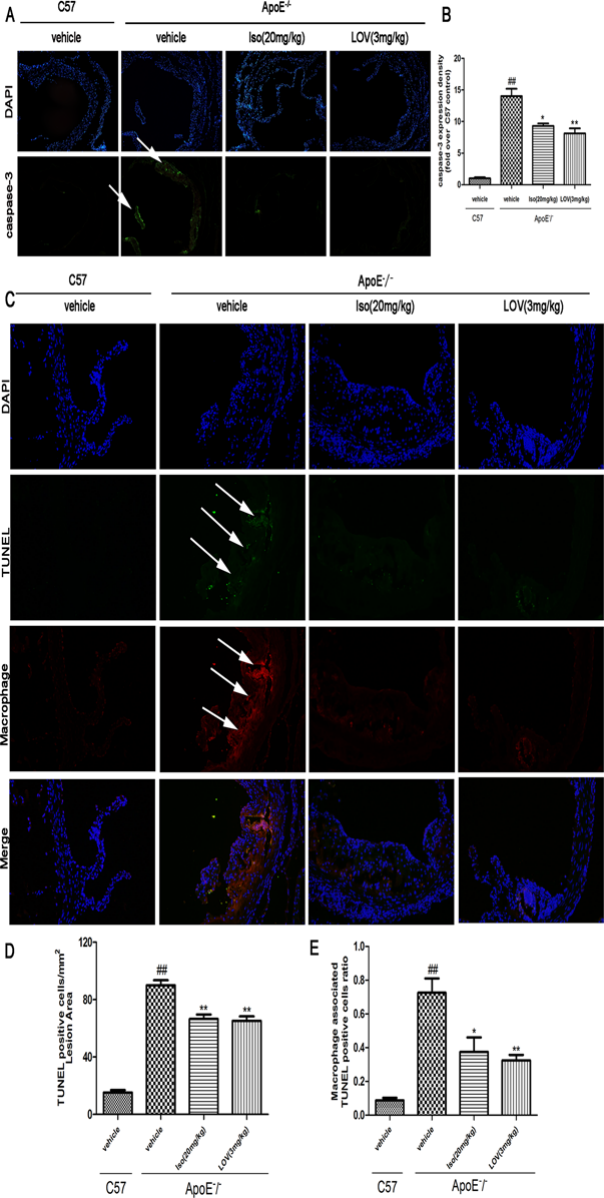
Iso showed inhibitory effects on macrophage apoptosis in atherosclerotic lesions. Mice were fed an HFC diet. ApoE-/- mice were treated with Iso (20 mg/kg, i.g.), LOV (3 mg/kg, i.g.), or its vehicle (as model group) for eight weeks; C57 mice were used as the control group. (A, B) Representative images of caspase-3 expression under immunofluorescent staining. (C) Representative images of the TUNEL assay. Macrophage nuclei (DAPI, blue), nuclei+TUNEL-positive staining (green), and merged images of macrophage cytoplasm (red). (D, E) Quantity of TUNEL-positive cells and ratio of macrophage-associated TUNEL-positive cells. Values (*n* = 10 per group) are expressed as the mean ± SD. ^*##*^ *P* < 0.01 compared with the C57 control group; *P < 0.05, **P < 0.01, compared with the vehicle-treated ApoE-/- model group.

Macrophage apoptosis has been shown to influences AS development [33]. We examined the development of AS in ApoE-/- mice. Following previously published methods, we stained macrophage cytoplasm and nuclei in apoptotic lesions to quantify the number macrophage-associated apoptotic bodies [27]. Iso administration was found to reduce caspase-3 expression compared with the model group (Fig. 9A and 9B). TUNEL staining showed that the model group contained significantly more apoptotic cells per mm^2^ lesion area compared with the control group. In contrast, a decreased number of apoptotic cells were observed in the lesions of the Iso and LOV groups compared with the lesions of model group (Fig. 9C, 9D, and 9E). These findings suggest that Iso could decrease the occurrence of macrophage-induced apoptosis in atherosclerotic lesions.

## Discussion

AS is a progressive inflammatory disease caused by multiple factors, one of which is macrophage-induced cell apoptosis [2]. Macrophage apoptosis caused by ox-LDL has recently attracted increased attention; however, no effective treatment has been developed. Iso is a flavonoid compound found in sea-buckthorn and traditionally used in Chinese medicine, and it has multi-bioactivities [18, 34]. This study evaluated the underlying mechanism of Iso on ox-LDL-induced macrophage injuries and anti-atherosclerosis. This is the first report of its kind to indicate that Iso may be a potential candidate in preventing AS.

Ox-LDL induces apoptosis in macrophages via oxidative stress injuries by promoting the high intracellular expression of ROS and NOX activity plays a critical role in ROS production [35]. In this study, we showed that Iso inhibited ox-LDL-induced ROS production and NOX activity in macrophages, which suggests the anti-oxidative activity of Iso. MPO and GSH-px are two enzymes that modulate free radicals. Our data show that Iso inhibited ROS production and MPO activity and increased GSH-px activity, corresponding to the observations of a previous study [36].

Ox-LDL-induced macrophage apoptosis is one of the factors leading to AS. Many anti-atherosclerotic therapeutics exert their atheroprotective effects by inhibiting macrophage apoptosis [28, 37]. Ning and colleagues demonstrated that inhibiting intracellular lipid levels protects cells from apoptosis [38]. Similarly, the present study showed that Iso suppressed lipid deposition in ox-LDL-treated THP-1-derived macrophages as revealed by oil red O staining. This finding suggests that Iso possessed an anti-atherosclerotic function by attenuating lipid deposition in ox-LDL-induced macrophage apoptosis.

Overproduction of ROS leads to cell apoptosis and mitochondrial dysfunction by oxidizing mitochondrial proteins, which results in further ROS production [39]. The damaged mitochondrial membrane hyperpolarization initiates the loss of MTP, the mitochondrial translocation of Bcl-2 and Bax, and cytochrome c release [40]. In accordance with previous reports [31], our data demonstrated that Iso could prevent ox-LDL-induced mitochondrial membrane potential damage. Iso pretreatment increased the expression of anti-apoptotic Bcl-2 but decreased the expression of pro-apoptotic Bax. Furthermore, Iso pretreatment suppressed Apaf-1 expression and thereby inhibited apoptosome formation and caspase-9 and caspase-3 activation. Caspase-3 activation is an initiative process of cell apoptosis. Activated caspase-3 induces DNA fragmentation and other morphological changes consistent with cell death [41]. Our study showed that Iso significantly suppressed ox-LDL-induced caspase-3 activation. We also found that Iso reduced the number of TUNEL-positive cells.

**Fig 10:**
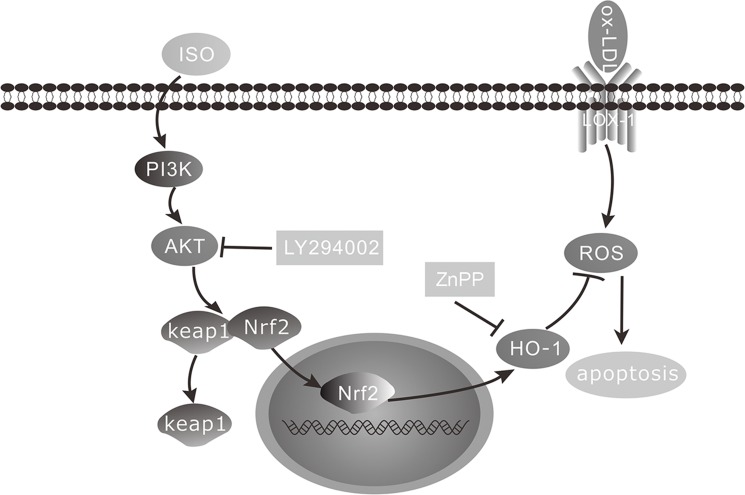
Schematic of Iso mechanism of preventing ox-LDL-induced apoptosis by activation of PI3K/AKT signaling and up-regulation of HO-1 in THP-1-derived macrophages.

HO-1, an endogenous defense enzyme, prevents oxidative stress injuries and is up-regulated by Nrf2 activation. Studies have shown that the PI3K/AKT pathway is involved in Nrf2 activation in macrophages [42]. The PI3K/AKT pathway is involved in protecting against not only apoptotic insults but also ROS detoxification [7]. It has been reported that Iso showed anticancer activity via derectly inhibiting PI3K [18]. However, in the present study, we demonstrated that Iso notably increased HO-1 expression, activated AKT phosphorylation, and promoted Nrf2 translocation. These results indicated that Iso showed selective effects on AKT from different cells. However, results showed that Iso has not obvious effects on other Nrf2 kinases (S1 Fig.). The addition of the AKT and HO-1 inhibitors may abolish the aforementioned effects of Iso. These results suggest that the anti-apoptotic effects of Iso were related to the activation of the PI3K/AKT pathway and HO-1 expression (Fig. 10).

To investigate the effect of Iso on the development of AS *in vivo*, ApoE-/- mice fed an HFC diet were studied. The ApoE-/- mouse model is well established in our laboratory for studies on AS [43]. In this study, we performed an oral administration of Iso (20 mg/kg, i.g.) and LOV (3 mg/kg, i.g.). After treatment for eight weeks, Iso reduced the atherosclerotic plaque size in the aortic valve and proximal aorta as assessed by oil red O and Sudan IV staining. CD68 staining indicated that Iso abolished macrophage accumulation in atherosclerotic lesions. Past studies have revealed that the accumulation of apoptotic macrophages results in the development of AS [44]. In agreement with our *in vitro* results, the *in vivo* mice models show that Iso reduced cell apoptosis in macrophage-enriched lesions by decreasing caspase-3 expression and the number of TUNEL-positive cells.

The lipid profile in mice plasma was evaluated. The results show that the concentrations of TG, TC, HDL and LDL were not significantly ameliorated by Iso treatment, thereby indicating that the atheroprotective effects of Iso were not due to the exclusion of a hypercholesterolemic source in ApoE-/- mice.

In conclusion, the results of this study suggest that Iso pretreatment significantly ameliorated ox-LDL-induced macrophage injuries. We also found that Iso activated the PI3K/AKT pathway, increased HO-1 production, and attenuated both ROS levels and lipid deposition in ox-LDL-treated THP-1-derived macrophages. Moreover, we found that Iso reduced atherosclerotic plaque sizes in ApoE-/- mice fed a Western diet as assessed by oil red O and Sudan IV staining. More importantly, Iso treatment reduced the accumulation of apoptotic macrophages in lesions. We identified a novel mechanism using Iso to prevent AS. Our results suggest that Iso may have potential therapeutic properties for the treatment of AS.

## Supporting Information

S1 Fig Iso has no effects on PKC and ERK kinases. Representative images of p-PKC, PKC, p-ERK1/2, ERK1/2 and β-actin expression levels and statistical results relative to control group. The blots are representative of three independent experiments, and values are presented as the mean ± SD from three independent experiments. ^*#*^ *P* < 0.05 *vs*. control.
(TIF)
