# Supplementary figures and images for: Isorhamnetin Attenuates Atherosclerosis by Inhibiting Macrophage Apoptosis via PI3K/AKT Activation and HO-1 Induction
Source: PLoS One. 2015 Mar 23;10(3):e0120259. doi: 10.1371/journal.pone.0120259 (PMC4370599; doi:10.1371/journal.pone.0120259)

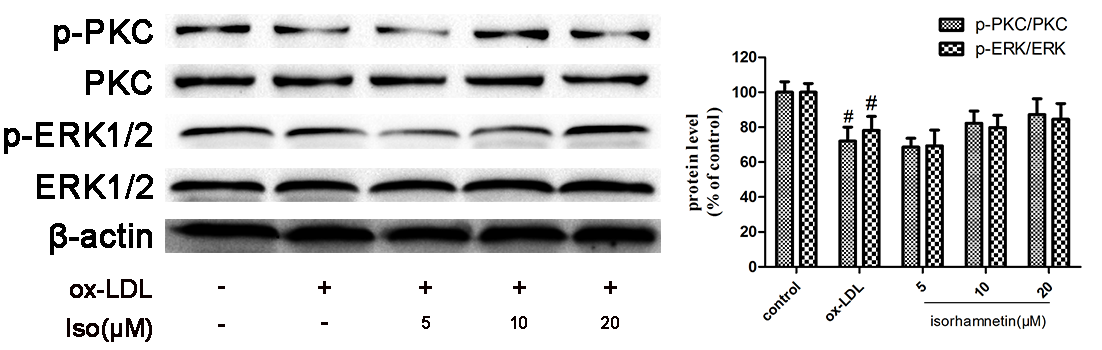

Supplement: S1 Fig — Representative images of p-PKC, PKC, p-ERK1/2, ERK1/2 and β-actin expression levels and statistical results relative to control group. The blots are representative of three independent experiments, and values are presented as the mean ± SD from three independent experiments. # P < 0.05 vs. control. (TIF) [file pone.0120259.s001.tif]
